# Operation Room Staff Professional Commitment and Related Factors at Amhara Referral Hospitals, Northwest Ethiopia, 2023; Multi‐Center, Cross‐Sectional Study

**DOI:** 10.1002/puh2.70155

**Published:** 2025-10-24

**Authors:** Alaye Debas Ayenew, Belete Muluadam Admassie, Tadesse Belayneh Melkie, Nurhusen Riskey Arefayne, Zewditu Abdissa Denu, Demelash Gedefaye Anteneh

**Affiliations:** ^1^ Department of Anesthesia College of Medicine and Health Sciences Debre Markos University Debre Markos Amhara Ethiopia; ^2^ Department of Anesthesia, College of Medicine and Health science Bahir Dar University Bahir Dar Amhara Ethiopia; ^3^ Department of Anesthesia College of Medicine and Health Sciences University of Gondar Gondar Amhara Ethiopia

**Keywords:** commitment, health professionals, occupational, operating room

## Abstract

**Background:**

Professional commitment is essential in high‐stress hospital environments like the operating room (OR), influencing healthcare quality, patient satisfaction, and outcomes. Poor commitment can lead to staff absenteeism, yet data on the commitment of OR staff and its influencing factors remain limited.

**Objective:**

To assess the professional commitment of OR staff and its associated factors at Amhara referral hospitals, Northwest Ethiopia, in 2023.

**Methods:**

A multi‐center, cross‐sectional study was conducted from April 20 to May 20, 2023, involving 424 participants selected through simple random sampling. Data were collected using a pretested, self‐administered questionnaire and analyzed using SPSS version 26. Factor analysis was performed after verifying assumptions. Simple and multiple linear regressions identified associated factors, with statistical significance set at *p* < 0.05 and a 95% confidence interval (CI).

**Results:**

The study achieved a 96.9% response rate. The mean professional commitment score was 67.4% (95%CI: 66.6, 71.0). Principal component analysis identified one factor explaining 57.1% of the variance in professional commitment. Significant predictors included educational level (*B* = 1.80; CI: 0.257, 3.350), monthly income (*B* = 1.89; CI: 0.324, 3.452), work experience (*B* = 1.51; CI: 0.284, 2.731), affective organizational commitment (*B* = 0.60; CI: 0.305, 0.821), normative organizational commitment (*B* = 0.25; CI: 0.120, 0.377), personal characteristics (*B* = 0.24; CI: 0.145, 0.342), and ethical leadership (*B* = 0.09; CI: 0.011, 0.163).

**Conclusion and Recommendation:**

Professional commitment among OR staff was moderate. Enhancing commitment requires targeted interventions such as training and educational opportunities, salary structure revisions, and the promotion of ethical leadership among healthcare managers and supervisors.

## 1 Introduction

### 1.1 Statement of the Problem

Commitment reflects an individual's connection to a goal, organization, or job and shapes their attitude toward work [1, 2, 3]. Professional commitment refers to a person's dedication to their profession, demonstrated by their willingness to work hard and uphold its values and objectives [4].

In healthcare, professional commitment is essential, as it encompasses adherence to ethical principles, effective communication among healthcare professionals, and continuous development of knowledge and expertise [4]. It significantly influences professional interactions and the quality of healthcare services. In particular, operating room (OR) staffs require strong professional commitment to maintain patient safety and deliver high‐quality care in their demanding and high‐risk work environment [5, 6].

The OR is one of the most challenging clinical settings in healthcare institutions [7]. Efforts to improve surgical outcomes focus on transparency, patient satisfaction, and reducing errors. However, human errors in ORs remain a leading cause of unintended injuries in hospitals due to their complex and high‐stress conditions [8, 9]. Globally, surgical system challenges persist [10, 11, 12]. To enhance surgical care, Ethiopia introduced the “Saving Lives Through Safe Surgery (SALT)” policy, making surgery a national priority [13].

Various factors influence health professionals’ commitment, including job status, salary, autonomy, training opportunities, workplace stress, and demographic factors [14, 15]. Enhancing job satisfaction and fostering a supportive work environment could strengthen OR staff commitment [16]. However, OR professionals often experience high stress, long working hours, sleep deprivation, and exposure to critical medical emergencies, increasing the risk of burnout and impaired surgical performance [17, 18, 19]. Studies indicate that burnout affects approximately 32.7% of Ethiopian health workers [20].

Professional commitment levels among healthcare workers vary globally. Studies indicate that nurses’ commitment ranges from 32% to 67% in high‐income countries [14, 21] and 55% to 86% in low‐ and middle‐income countries [4, 22]. In Ethiopia, healthcare providers involved in institutional deliveries report professional commitment levels of 70%–73% [23, 24]. However, the Ethiopian healthcare system faces persistent challenges, including a shortage of motivated healthcare workers and frequent absenteeism in public hospitals, which may be linked to low professional commitment [25, 26]. A lack of commitment among healthcare professionals can compromise patient safety, increase medical errors, and ultimately weaken the healthcare system [14, 27].

Despite existing research on healthcare professional commitment in Ethiopia, studies on OR staff commitment remain limited. Given the unique challenges faced by OR personnel, further investigation is necessary. This study aims to explore the professional commitment of OR staff, including nurses, anesthetists, and surgeons, in northwest Amhara referral hospitals and identify key contributing factors.

### 1.2 Methodology

Before conducting the study, ethical clearance and approval were obtained from the Institutional Review Board (IRB) of the College of Medicine and Health Sciences, University of Gondar (Ref. No. SOM 557/2023). Additionally, permission was secured from the respective hospitals. Each questionnaire was accompanied by a letter of consent outlining the study's objectives and providing further details. To ensure anonymity and confidentiality, participants’ names were replaced with unique codes. Furthermore, oral informed consent was obtained from all participants before administering the questionnaires.

### 1.3 Study Design, Study Setting, and Population

This multi‐center, cross‐sectional study was conducted in the northwest Amhara National Regional State, Ethiopia. The study included five comprehensive specialized hospitals:

- University of Gondar Comprehensive Specialized Hospital (UOGCSH)
- Tibeb Gihon Specialized Hospital (TGSH)
- Felege Hiwot Referral Hospital (FHRH)
- Debre Tabor Comprehensive Specialized Hospital (DTCH)
- Debre Markos Comprehensive Specialized Hospital (DMCSH)

Eligible participants included OR staff with a minimum of 6 months of experience, actively involved in surgical, anesthetic, or nursing care. Individuals who were critically ill, on annual leave, or in nonclinical roles were excluded.

### 1.4 Operational Definitions

Professional commitment: It is the degree to which a person is connected to a given profession. It suggests the person's attitude toward their career and the drive they have to stay in their position, which is a reference to their commitment to the profession and their readiness to work hard and preserve its principles and aims. This was measured using 10 items on a 5‐point Likert scale, 1 denoting strongly disagree and 5 denoting strongly agree, which was adapted from a different study. A professional commitment score was created, and a higher score indicates higher professional commitment. The mean score for the scale was standardized as the percentage of the scale mean score (%SM) to facilitate comparison. This enables future researchers to easily compare their findings with those in this study even if they make use of a different number of items. This ranges from “0%” to “100%.” The mean percentage of the score was calculated as follows [23, 24, 28]:

$$\%\mathrm{SM}=\frac{\mathrm{Actual}\;\mathrm{score}-\mathrm{Scale}\;\mathrm{minimum}\;\mathrm{score}}{\mathrm{Scale}\;\mathrm{maximum}\;\mathrm{score}-\mathrm{Scale}\;\mathrm{minimum}\;\mathrm{score}}\times 100\%$$

### 1.5 Data Collection Procedure and Measurement

The data were collected using a pretested Likert scale‐type self‐administered English version questionnaire that was adapted from different literature and translated into Amharic, then back to English. The questionnaire has seven parts. Part I: participant characteristics (age, gender, marital characteristics, education level, salary, employment type, length of service). Part II: Professional commitment with 10 items, and the participants were asked to rate each item on a 5‐point Likert scale, which range from strongly disagree (1) to strongly agree (5) [24, 29]. Part III: Organizational commitment with 16 items, and the participants were asked to rate each item on 5‐point Likert scale, which range from strongly disagree (1) to strongly agree (5) [30].

Part IV: Work‐related stress with 23 items [31]. Part V: personal characteristics with 10 items [32]. Part VI: Ethical leadership with 10 items, and the participants were asked to rate each item on 5‐point Likert scale on 5‐point Likert scale, which ranges from strongly disagree (1) to strongly agree (5) [33]. Part VII: Work satisfaction with 15 items, the participants were asked to rate each item on 5‐point Likert scale on 5‐point Likert scale, which ranges from strongly disagree (1) to strongly agree (5) [34].

Two qualified anesthetists were assigned in each referral hospital, in which the first one collects data and the other supervises the data collection process who were given a 1‐day training to familiarize themselves with data collection procedure and instruments.

### 1.6 Sample Size Determination

The sample size was calculated by using the single population proportion formula as follows:

$$n=\left({\left(Z\;a/2\right)}^{2}\times \left(p\right)\times \left(q\right)\right)/d^{2}$$

where *n* is the minimal sample size, *𝑍* is the reliability coefficient, *a* is the critical value at 95% confidence interval (CI) of certainty (1.96), *d* is the marginal error/degree of precision = 5% (0.05), *p* = 0.7271. We take percentage of professional commitment toward institutional delivery service (72.71%) in the study conducted in Jimma zone southwest Ethiopia:

$$\begin{array}{c} \begin{array}{c} n=1.96\left(0.7271\right)\;\left(0.2729\right)/0.05\\ n=0.7623/0.0025 \end{array}\\ n=305\;\mathrm{with}\;10\%\;\mathrm{nonresponse}\;\mathrm{rate}\\ n=336 \end{array}$$

Sample size calculation for factors with the following assumptions: 95%CI, 80% power, and one‐to‐one unexposed to the exposed ratio (a study done in Shone District, Southern Ethiopia, shows that organizational commitment and personal characteristics are associated with an increase in the level of professional commitment with a *p* value <0.001).

The sample size calculated from the secondary objectives was less than that calculated from the dependent variable. By considering statistical analysis method used and to increase the generalizability of study, we increase the sample size to 424 (Table 1).

**TABLE 1:** Sample size calculation by factors from a study of professional commitment and associated factors toward institutional delivery service providers in Shone District, Southern Ethiopia, 2023.

Variables	% Outcome in exposed groups	% Outcome in unexposed groups	AOR	Sample size
Organizational commitment	77.9	59.0	2.27	210
Personal characteristics	77.4	53.4	1.76	204

### 1.7 Sampling Procedures and Techniques

**FIGURE 1:**
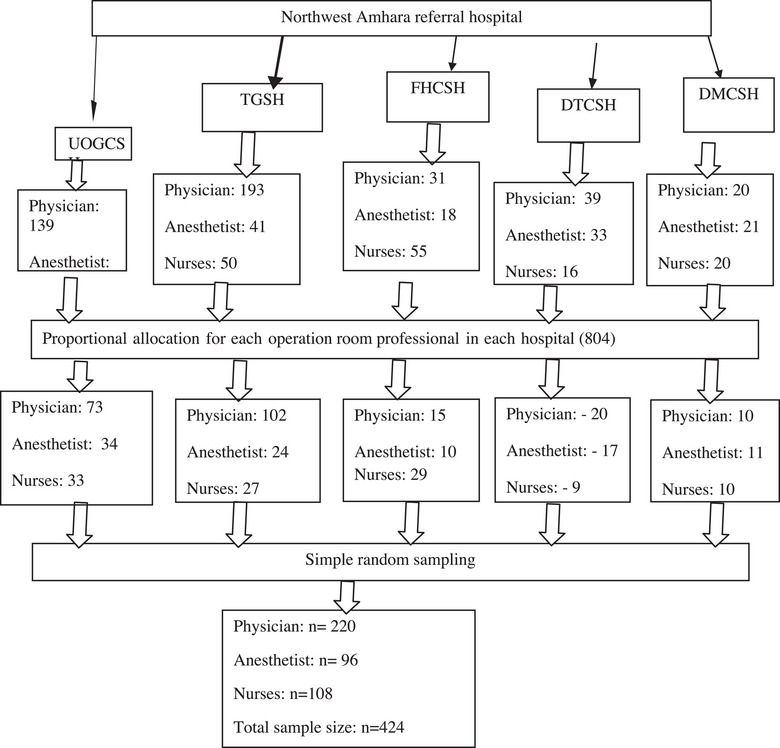
Schematic presentation of the sampling procedure for study on the professional commitment and its associated factors among operation room staff working in northwest Amhara region, Northwest Ethiopia, 2023. DMCSH, Debre Markos Comprehensive Specialized Hospital; DTCSH, Debre Tabor Comprehensive Specialized Hospital; FHCSH, Felege Hiwot Referral Hospital; TGSH, Tibeb Gihon Specialized Hospital; UOGCSH, University of Gondar Comprehensive Specialized Hospital.

A simple random sampling technique was employed to select study participants, ensuring proportional representation from each of the five referral hospitals based on the number of eligible professionals in each facility (Figure 1).

### 1.8 Data Quality Management

To ensure data quality, the questionnaire was pretested on 5% of the actual sample size 1 week before data collection. On the basis of the pretest results, necessary modifications were made. Additionally, data collectors received 1 day of training, and data completeness was checked daily. Incomplete questionnaires were discarded during data entry. To minimize entry errors, EPI DATA version 4.6 was used. Data cleaning involved proper categorization and coding to ensure accuracy.

### 1.9 Data Management and Processing

After data collection, the principal investigator manually reviewed the questionnaires for completeness and consistency. Each completed questionnaire was assigned a unique identification code, and negatively worded items were reverse‐coded to ensure consistency in analysis. The data were then entered into EpiData version 4.6 to minimize entry errors. After verification, the dataset was exported to SPSS version 26 for statistical analysis.

### 1.10 Data Exploration and Assumption Testing

The dataset was explored using descriptive statistics and frequency distributions to ensure data quality and cleanliness. Homoscedasticity was assessed using scatter plots of residuals, confirming that the residuals maintained a consistent variance across predicted values. Normality of the data distribution was examined using histograms, which indicated a normal distribution. On the basis of these assessments, no data transformations were required. Multicollinearity was checked by calculating the variance inflation factor (VIF) for each independent variable. As all VIF values were ≤10, multicollinearity was not a concern, and all variables were retained in the model. These tests ensured that the data met key assumptions for multiple regression analysis.

### 1.11 Data Analysis

Factor analysis was conducted to identify key factors related to the professional commitment scale and other independent variables. Before performing principal component analysis (PCA), the suitability of the data for factor analysis was assessed. The results indicated the presence of many coefficients ≥0.5, a Kaiser–Meyer–Olkin (KMO) measure of sampling adequacy >0.88, and a significant Bartlett's test of sphericity (*p* < 0.001). These findings confirmed that the data met the necessary assumptions for factor analysis.

To determine the number of factors to retain, the following criteria were applied:

- Eigen value ≥1 for each factor.
- Factor loadings ≥0.5 for individual items.
- At least three items loading onto each factor, as factors with fewer than three items were considered weak and unstable.
- Cronbach's alpha ≥0.70 to ensure internal reliability.
- The scree plot test, retaining all factors above the “elbow” point, as they contributed the most to explaining variance in the dataset.

For factor extraction, varimax rotation was applied to minimize cross‐loading and enhance factor interpretability.

### 1.12 Regression Analysis

Before performing linear regression, categorical variables were converted into dummy variables. Bivariate correlation analysis was conducted to assess relationships between independent and dependent variables. Variables with a significant correlation (*p* < 0.005) were considered candidates for regression analysis.

In the bivariate analysis, variables with a *p* value <0.2 were entered into the final multiple linear regression model. Factors associated with professional commitment were identified using multiple linear regression analysis at a significance level of *p* < 0.05 with a 95%CI. The adjusted *R* ^2^ was used to assess the strength of association in the final model.

Finally, the results were summarized and presented in tables and descriptive statements for interpretation.

## 2 Results

### 2.1 Characteristics of the Study Participants

A total of 424 participants were included in the study, with 411 (96.9%) completing the questionnaires. Of these, 294 (71.5%) were male, and 229 (55.7%) were married. Regarding professional roles, 207 (50.4%) were physicians, and 94 (22.9%) were anesthetists. In terms of experience, 232 (56.4%) had 2–5 years of experience. The median age of participants was 30 years (IQR: 28–33). In terms of educational background, 155 (37.7%) held a Bachelor of Science degree, whereas 247 (60.1%) had postgraduate degrees (Table 2).

**TABLE 2:** Socio‐demographic characteristics of operation room staff in northwest Amhara referral hospitals, Northwest Ethiopia, 2023 (*n* = 411).

Variables		Frequency (*n*)	Percentage
Sex	Male	294	71.5
	Female	117	28.5
	20—24	2	0.5
Age in years	25–29	176	42.8
	30–34	175	42.6
	>34	58	14.1
	Single	178	43.3
Marital status	Married	229	55.7
	Divorced/Windowed	4	1
	Diploma	9	2.2
Educational status	1st degree	155	37.7
	2nd degree and above	247	60.1
	Anesthetist/Anesthesiologist	94	22.9
Profession	Physician	207	50.4
	Nurse	110	26.7
	<2 years	20	4.9
Work experience in years	2–5 years	232	56.4
	>5 years and above	159	38.7
	≤6193	61	14.8
Average monthly incomes	6194–7071	47	11.4
	7072–8017	36	8.8
	8018–9056	26	6.3
	9057–10, 150	33	8
	>10, 150	208	50.6
Status of employment in the hospital	Academician	182	44.3
	Clinician	229	55.7

### 2.2 Magnitude of Professional Commitment

Among the 411 study participants, the overall professional commitment of OR staff was 67.4%, with a 95%CI of 66.6%–71.0%. The mean professional commitment score was 37.5 ± 8.8, with 57.1% of the total variance explained.

Out of 411 study participants, approximately 31.2% agreed, and 32.1% strongly agreed with the idea that they were willing to put in a great deal of effort to develop their profession. Additionally, about 28.2% agreed, and 34.8% strongly agreed with the perception that they were proud to belong to their profession.

Of the 411 study participants, more than one‐third (34.3%) agreed, and nearly one‐third (26.5%) strongly agreed with the idea that they were inspired to give their very best in their job performance. Likewise, 31.4% agreed, and 41.1% strongly agreed with the notion that they considered their profession important. However, only 9.2% agreed, and 5.1% strongly agreed with the perception that they were criticized within their profession (Table 3).

**TABLE 3:** Frequency distribution of the 5‐point Likert scale response of professional commitment measuring items among operation room staff working in northwest Amhara referral hospital, Ethiopia, 2023 (*n* = 411).

Items measuring professional commitment	Strongly disagree	Disagree	Neutral	Agree	Strongly agree
I am willing to put in a great deal of effort to develop my profession beyond expected	26 (6.3%)	45 (10.9%)	79 (19.2%)	132 (32.1%)	129 (31.4%)
I am a person who identifies strongly with my profession	25 (6.1%)	42 (10.2%)	75 (18.2%)	139 (33.8%)	130 (31.6%)
I would accept almost any type of job that related to my profession to keep working beyond expected from me	13 (3.2%)	60 (14.6%)	83 (20.2%)	134 (32.6%)	121 (29.4%)
I am a person who feels strong ties with other members of my profession	16 (3.9%)	40 (9.7%)	76 (18.5%)	156 (38%)	123 (39.9%)
I am a person who is proud to belong to my profession	22 (5.4%)	50 (12.2%)	80 (19.5%)	116 (28.2%)	143 (34.8)
My profession really inspires the very best in me in the way of job performance	26 (6.3%)	56 (13.6%)	79 (19.2%)	141 (34.3%)	109 (26.5%)
I am extremely glad that I chose this profession to work forever in advance	29 (7.1%)	60 (14.6%)	99 (24.1%)	102 (24.8%)	121 (29.4)
I am a person who criticizes my profession	159 (38.7%)	124 (30.2%)	69 (16.8%)	38 (9.2%)	21 (5.1%)
I am considering my profession to be important	25 (6.1%)	20 (4.9%)	68 (16.5%)	130 (31.6%)	168 (40.9%)
I am a person who tries to hide belonging to my profession	154 (37.5%)	124 (30.2%)	65 (15.8%)	45 (10.9%)	23 (5.6%)

### 2.3 Descriptive Statistics of Factors Emerged From Factor Analysis of Independent Variables

Factor analysis using principal factor analysis was conducted to determine the number of factors extracted and the total variability explained. Three factors were identified to represent perceived organizational commitment. Among these, normative commitment had the highest mean score percentage at 58.6%, whereas continuous commitment had the lowest at 48.8%. Together, these three factors explained a total of 60.89% of the variability, with a reliability coefficient of 0.91.

For work‐related stress, five factors were initially assessed, but two were excluded from further analysis due to a reliability coefficient of less than 0.7. The factor with the highest mean score was social support at work (61.7%), whereas work‐life balance had the lowest mean score at 44.7%. Overall, the remaining factors explained a total of 64.8% of the variability.

The personal characteristics of OR staff had a percentage mean score of 65.5%, with a mean raw score of 36.16 ± 7.9. The model explained a total variance of 50.3%, with a reliability coefficient of 0.89. The OR staff's perceived ethical leadership had a mean raw score of 32.4 ± 9.7, and the percentage mean score was 56%. The model accounted for a total variance of 63.8%, with a reliability coefficient of 0.94.

Job satisfaction was influenced by three factors, with staff relationships receiving the highest percentage mean score of 56.9%. Perceived benefits/recognition had the lowest percentage mean score at 37.7%. The model explained a total variability of 64.4% (Table 4).

**TABLE 4:** Summary of scales emerged from factor analysis and a mean score of predictors of professional commitment among operation room staff working in northwest Amhara referral hospital, Northwest Ethiopia, 2023 (*n* = 411).

	Emerged factors	%SM	Mean raw score ± SD	No. item	Cronbach's alpha: >0.7	Total variability (%)
	Normative commitment	58.6	23.4 ± 7	7	0.89	60.88
**Organizational commitment**	Affective commitment	55.6	12.9 ± 4.4	5	0.87	
	Continuance commitment	48.8	11.8 ± 3.2	4	0.71	
**Work‐related stress**	Psychological demand	44.7	25.1 ± 8.4	9	0.89	64.76
	Role expectation conflict	48.3	8.8 ± 2.8	3	0.79	
	Social support at work	61.8	13.9 ± 3.75	4	0.86	
	Work life balance at work	57.5	16.5 ± 4.86	5	0.83	
	Lack of resource 53%		6.16 ± 1.9	2	0.63	
**Personal characteristics**	Personal characteristics	65.5	36.16 ± 7.9	10	0.89	50.3
**Ethical leadership**	Ethical leadership	56	32.4 ± 9.7	10	0.94	63.8
**Job satisfaction**	Autonomy at work	53.3	9.4 ± 3	3	0.84	64.4
	Staff relationship	56.9	13.1 ± 3.8	4	0.81	
	Perception on benefit/recognition from manager	37.7	19.3 ± 7.4	8	0.89	
**Professional commitment score**		67.4 CI (66.6, 71.0)	37 ± 8.8	10	0.91	57.1

Abbreviations: %SM, percentage of scale mean score; CI, confidence interval; SD, standard deviation.

### 2.4 Factors Associated With Professional Commitment

This study employed a bivariate linear regression model to analyze several demographic factors, including age, education level, experience, and net monthly income. Among these variables, age (*p* value: 0.139), experience (*p* value: 0.13), education level (*p* value: 0.01), and net monthly income level (*p* value <0.001) were found to be significant and were included in the multiple linear regression analysis. The socio‐demographic predictors accounted for only 18% of the variability in the professional commitments of the study participants (*R*: 0.451, *R* ^2^: 0.204, adjusted *R* ^2^: 0.180).

Three factors related to organizational commitment were included in the model, and all were found to be significant factors of professional commitment. These three factors explained 47.2% of the variability in professional commitment scores.

Three factors related to work‐related stress were entered into the bivariate linear regression model. Psychological demands (*p* value <0.001), work‐life balance (*p* < 0.001), and social support at work (*p* value <0.001) were significant, whereas the factors related to lack of resources (*p* value: 0.903) and ambiguity in role expectations (*p* value: 0.734) were not significant. The model explained 25.7% of the variability.

Personal characteristics scores (*p* value <0.001) were significant factors in the professional commitment of OR staff, explaining 36% of the variability in professional commitment (*R*: 0.601, *R* ^2^: 0.361). Ethical leadership scores (*B*: 0.416, *p* value <0.001) were also significant, accounting for 20.6% of the variability in professional commitment.

Three factors related to job satisfaction were entered into the bivariate linear regression model: autonomy, staff relationships, and perceived benefits/recognition. These factors were significant and were included in the multiple linear regression analysis. The perceived job satisfaction factors explained 26.3% of the variability in professional commitment among OR staff (Table 5).

**TABLE 5:** Factors related to professional commitment among operating room staff working in northwest Amhara referral hospital, Northwest Ethiopia, 2023 (*n* = 411) (bivariate linear regression).

					95%CI for *B*	
Variables		*B*	Adjusted *R* square	*p* value	Lower bound	Upper bound
Age	25–29	3.02	0.180	0.626	−9.15	15.198
	30–34	3.70		0.540	−8.37	15.973
	>34	8.08		0.197^**^	−4.22	20.400
Education level	Postgraduate	6.37		0.06^**^	1.66	20.400
	Degree	0.85		0.725	−3.92	20.400
Experience	>5 year	3.1		0.127^**^	0.887	0.089
	>2–5 years	0.265		0.897	−3.794	4.325
Income level	7072–8017	0.136		0.936	−3.221	3.49
	6194–7071	1.505		0.340	−1.595	4.60
	8018–9056	0.495		0.794	−3.246	4.2
	9057–10, 150	2.04		0.244	−1.407	5.49
	>10, 150	7.85		0.000^**^	5.529	10.18
OC	Affective commitment	1.333	0.472	0.000^**^	1.185	1.48
	Continuous commitment	0.921		0.000^**^	0.672	1.17
	Normative commitment	0.769		0.000^**^	0.671	0.866
WS	Psychological demand	−0.227	0.257	0.000^**^	−0.327	−0.126
	Work life balance	0.731		0.000^**^	0.569	0.893
	Social support at work	0.951		0.000^**^	0.742	1.160
	Factors related to lack of resources	−0.019		0.901	−0.329	0.290
	Ambiguity in role expectation	0.078		0.734	−0.374	0.530
PC	Personal characteristics	0.672	0.359	<0.001^**^	0.585	0.759
EL	Ethical leadership	0.416	0.206	<0.001^**^	0.337	0.494
JS	Autonomy at work	1.101	0.263	<0.001^**^	0.836	1.365
	Staff relationship	1.105		<0.001^**^	0.908	1.302
	change of getting benefit/recognition	0.448		<0.001^**^	0.341	0.555

Abbreviations: EL, ethical leadership; JS, job satisfaction; OC, organizational commitment; PC, personal characteristics; WS, work stress.

** Significant at *p* value <0.2 (candidate for MLR).

### 2.5 Independent Predictors of OR Professional Commitment

The backward conditional model in multiple linear regression identified seven significant factors of professional commitment, which accounted for 55.5% of the variability in professional commitment scores (*R*: 0.752, *R* ^2^: 0.566, adjusted *R* ^2^: 0.555).

Among the socio‐demographic variables, work experience, educational level, and monthly salary were found to be significant factors of professional commitment. Specifically, having more than 5 years of work experience resulted in a 1.5 U higher professional commitment score compared to having less than 2 years of experience, while holding all other variables constant. Individuals with a postgraduate degree had a 1.8 U higher professional commitment score compared to those with a diploma, again holding all other variables constant. Additionally, individuals with a monthly salary greater than 10, 150 Ethiopian Birr had a 1.9 U higher professional commitment score compared to those earning less than 6169 Ethiopian Birr, whereas all other variables were held constant.

Among the significant predictors of organizational‐related factors, affective organizational commitment showed a positive relationship with professional commitment scores. We are 95% confident that a 1‐U increase in the affective organizational commitment score of OR staff leads to a 0.6 U increase in their professional commitment score (*p* value <0.001, 95%CI: 0.38, 0.82), while keeping all other variables constant. Furthermore, normative organizational commitment also showed a positive relationship with professional commitment scores. A 1‐U increase in the normative organizational commitment score of OR staff results in a 0.25 U increase in their professional commitment score (*p* value: 0.000, 95%CI: 0.12, 0.38), with all other variables held constant.

Other predictors of professional commitment scores included perceived personal characteristics and perceived ethical leadership. A 1‐U increase in the perceived personal characteristics score results in a 0.24‐U increase in the professional commitment score of OR staff (*p* value <0.001, 95%CI: 0.14, 0.34). Additionally, a 1‐U increase in the ethical leadership score leads to a 0.09‐U increase in the professional commitment score (*p* value: 0.025, 95%CI: 0.01, 0.16), while holding all other variables constant (Table 6).

**TABLE 6:** Independent predictors of professional commitment among operating room staff in northwest Amhara referral hospital, Northwest Ethiopia, 2023 (*n* = 411) (multivariable linear regression).

	Unstandardized coefficients	Standardized coefficients		95.0%CI for *B*	
Independent predictors	*B*	Beta	*p* value	Lower bound	Upper bound
Affective commitment	0.603	0.297	0.000^**^	0.305	0.821
Continuous commitment	0.190	0.99	0.055	−0.004	0.384
Normative commitment	0.248	0.65	0.000^**^	0.120	0.377
Personal characteristics	0.243	0.05	0.000^**^	0.145	0.342
Ethical leadership	0.087	0.039	0.025^*^	0.011	0.163
Change of getting benefit/recognition	0.096	0.050	0.058	−0.003	0.195
Age >34	1.25	0.639	0.050	−0.003	2.507
Postgraduate	1.804	0.787	0.022^*^	0.257	3.350
Experience >5 year	1.507	0.622	0.016^*^	0.284	2.731
Income level >10, 150	1.889	0.786	0.018^*^	0.324	3.452

*Note:* *R*: 0.752, *R* square: 0.566, adjusted *R* square: 0.555, Durban Watson test: 1.78, Max VIF: 3.1.

* Significant at *p* value <0.05.

** Significant at *p* value <0.001.

## 3 Discussion

This study finding revealed that the professional commitment score of the OR staff was 67.4%, with a 95%CI (66.6, 71.0). This finding was lower than another studies that reported in India and Jimma zone, Ethiopia, that the level of commitment mean scores were 85.9% and 72.71%, respectively [4, 24]. The variation may be attributed to the perks offered by the Indian organization, such as pensions, home and car loans, and medical benefits. Additionally, most of the respondents were doctors who exhibited heightened professional commitment through favorable rapport with their superiors, well‐defined work goals, and autonomy in their tasks. In the case of the study done in Ethiopia, large sample size and population were different.

This finding was higher than studies done in Nigeria, Saudi Arabia, and South West Ethiopia on nurse's professional commitment mean scores, which were 55.15%, 57.43%, and 36.3%, respectively [16, 35, 36]. In the case of a study done in southwest Ethiopia, the discrepancy may be due to profession type and small sample size. In the case of Saudi Arabia, the participants were only nurses, with a high workload and stress than in our study, which include nurses, physicians, and anesthetists. The situation in Nigeria could be connected to the payment that different health professions get. This research finding is in‐line with a study done in Shone District, which revealed that professional commitment scores were 69.4% [25]. The similarity may be related to the benefit offered by organization.

This study found that educational level 95%CI (0.251, 3.350) (monthly salary >10, 150 Ethiopian Birr), years of experience, and monthly income level are positive associate with professional commitment among socio‐demographic variables. This finding is consistent with another study [37], which found that there was an association between educational levels on professional commitment. This finding is also consistent with another study [34] that found health professionals having postgraduate study have a higher level of professional commitment compared to those with having diploma education level. The possible explanation might be related to the health profession having a high level of education level, which might have high salaries, benefits, and higher positions in the organization. In contrast to this, another study in China [38] found that educational level was not a significant factor in professional commitment. Therefore, education level influences professional commitment by enhancing knowledge, opening opportunities for advancement, reinforcing professional identity, and improving the quality of care provided.

The discrepancy in the case of China may be related to different populations and cultures. Years of experience, with 95%CI (0.284, 2.731), are positively associates with professional commitment in our study. This finding is supported by a study done in Ghana [22]. The possible explanation may be that health professionals having long years of experience have higher positions, wages, and benefits, and greater achievements. But not consistent with a study in China [38] that found there was no correlation between years of experience and professional commitment. The difference could be the study population difference and the small sample size in the case of China. Therefore, years of experience in the OR contribute to professional commitment by enhancing competence and confidence, leading to greater satisfaction; fostering job stability and long‐term loyalty to the organization; offering opportunities for leadership roles and mentoring, which reinforces commitment; encouraging ongoing professional development and growth; and even building respect and social capital within the team.

Income level (monthly salary >10, 150 Ethiopian birr), with 95%CI (0.324, 3.452), was significantly associated with professional commitment in our study. This study was in‐line with another study [39]. A possible explanation could be that increased income level may increase job satisfaction of health professions. However, in a study done in Ghana, income level was not a significant predictor of professional commitment [22]. The discrepancy could be the study population difference and the payment provided by Ghana health organizations. Therefore, monthly **s**alary affects professional commitment by ensuring financial security, recognition of expertise, and overall job satisfaction.

In this study, an association was found between affective organizational commitment and professional commitment to the health profession. A 1 U increase in affective organizational commitment was an increase of 0.6 U (95%CI: 0.305, 0.821) of professional commitment to the health profession. This can be explained by the health profession having affective organizational commitment high chance of emotional attachment, loyalty, a willingness to put in extra effort, and a sense of belonging and identification with the organization's goals and values. This may make health professionals committed to their profession. This finding is almost in‐line with a study done in India that showed that affective organizational commitment is positively associated with professional commitment [4]. Our finding is also consistent with the finding of a study done in southern Ethiopia that showed that affective commitment is positively associated with professional commitment [25]. Therefore, for OR staff, fostering **affective commitment** through positive organizational culture, leadership, professional development opportunities, and aligning values can significantly enhance their **professional commitment**, leading to better patient care, improved team dynamics, and greater satisfaction within the workplace.

In addition, in this study, normative organizational commitment with 95%CI (0.120, 0.377) was positively associated with professional commitment. Normative organizational commitment refers to an employee's sense of obligation or duty to remain with their organization. It is based on a belief that staying with the organization is the right thing to do and a desire to maintain one's professional reputation. Our finding is comparable to a study done in India [4] and Ethiopia [24]. In both study areas, those health professions who have a better level of normative organizational commitment have a better level of professional's commitment. The similarity may result from the connection between an individual's dedication to their organization and professional commitment, regardless of where in the world they are.

A significant association was found between posetive personal charactersitics and proffesional commitment.This midiated by individual capacity for detail orintation,leading to superior ethical conduct and effective intraction with patient and peers. Our finding is consistent with a study done in Ethiopia, which states that health workers with a good attitude about their personalities are more likely to be committed to their work than those with a negative attitude [24]. This finding is also consistent with a study done in New York, USA, on physician professional commitment, which found that physicians who believed in their autonomy and had a positive attitude toward their work were more committed to it [40]. Therefore, perceived personal characteristics affect professional commitment by influencing individual behaviors, attitudes, and motivations. Traits, such as self‐motivation, work ethic, personal values, resilience, emotional intelligence, professional identity, and self‐efficacy, contribute to a stronger sense of commitment to one's profession. In the OR, these characteristics lead to a more dedicated, responsible, and ethical workforce; this enhances both individual performance and overall patient care.

This study showed that ethical leadership style was a positive predictor of professional commitment. A unit increment of ethical leadership was 0.1 U (95%CI: 0.011, 0.163) increment in professional commitment to the health profession. This can be explained by the ethical leadership and ethical environment in the hospital that, in addition to providing a proper context for the ethical performance of health profession and staff, is positively important to promote professional commitment. Ethical leadership can also promote a culture of transparency and accountability.

This finding is similar when compared with a study done in Iran, which showed that a unit increment of ethical leadership was 0.77 U of the increment of professional commitment of the health profession [41]. The difference could be due to leadership style differences, large sample sizes, and tools used to measure ethical leadership in Iran. This finding was also supported by another study that states that when 1 U increment in ethical leadership, employee commitment was increased by 1.047 U. The discrepancy may be related to population differences; they investigate only in nonacademic staff. Therefore, ethical leadership influences **professional commitment** by fostering an environment of trust, fairness, respect, and integrity. Ethical leadership leads to clearer expectations, better job satisfaction, higher morale, and greater organizational loyalty; these might contribute to stronger professional commitment. For OR staff, ethical leadership can create an atmosphere where ethical decision‐making, high standards of patient care, and teamwork thrive, ultimately leading to higher levels of commitment to the profession.

## 4 Limitation of the Study

Although your study provides valuable insights into the **factors associated with professional commitment** among OR staff, it is limited by the **cross‐sectional design**, which prevents causal inferences. Additionally, there could be potential **information bias** in the data collection process, which may affect the accuracy of the findings. Future research, using more rigorous designs, would be necessary to confirm these relationships and explore cause‐and‐effect dynamics.

## 5 Conclusion

This study reveals that the professional commitment of OR staff is moderate, with a score of 67.4% among the 411 participants. The study also highlighted key factors such as work‐related stress, job satisfaction, and personal characteristics that contribute to the variability in professional commitment. Several independent predictors, including work experience, education level, and monthly income, with work experience of over 5 years, a postgraduate degree, and higher salary levels associated with higher professional commitment scores. Furthermore, affective and normative organizational commitments, along with ethical leadership, were significant positive factors influencing commitment. These findings underscore the importance of fostering a supportive work environment, providing opportunities for professional development, and promoting ethical leadership to improve the professional commitment of OR staff.

## 6 Recommendation

### 6.1 For Hospital Administrators

- Recognize that the current level of OR staff commitment remains unsatisfactory and take proactive measures to improve it.
- Invest in professional development opportunities such as ongoing training, skill enhancement programs, and continuing education.
- Foster a positive work environment by promoting teamwork, reducing workplace stress, and ensuring a supportive organizational culture.
- Offer competitive salaries and benefits to enhance job satisfaction and reduce staff turnover.
- Strengthen organizational commitment by increasing awareness of its importance and integrating it into hospital policies.
- Prioritize ethical leadership development among managers and supervisors to build trust and motivation among OR staff.

### 6.2 For OR Staff

- Actively work on personal and professional development to strengthen commitment to their roles.
- Cultivate ethical behavior and professionalism to enhance the quality of surgical services and patient care.
- Engage in teamwork and effective communication to contribute to a safer and more efficient OR environment.

### 6.3 For Researchers

- Conduct further studies exploring the relationship between professional commitment and professional communication among OR staff.
- Investigate additional factors, such as workplace culture, leadership styles, and psychological well‐being, that may influence OR staff commitment.

## Author Contributions

All authors made a significant contribution to the work reported, whether that is in the conception, study design, execution, acquisition of data, analysis and interpretation, or in all these areas; took part in drafting, revising, or critically reviewing the article; gave final approval of the version to be published; have agreed on the journal to which the article has been submitted; and agree to be accountable for all aspects of the work.

## Funding

The authors have nothing to report.

## Ethics Statement

Ethical approval was obtained from the Institutional Review Board (IRB) of the College of Medicine and Health Sciences, University of Gondar (Ref No. SOM 557/2023). Additionally, permission was secured from the respective hospitals. Each questionnaire was accompanied by a consent letter outlining the study's objectives and providing further details. To ensure anonymity and confidentiality, participants’ names were replaced with codes.

## Consent

A letter of consent outlining the aim and giving further details about the study accompanied each questionnaire. To assure anonymity and confidentiality, the name of the participant was replaced by a code. Furthermore, oral informed consent was obtained from all participants before administering the questionnaires.

## Conflicts of Interest

The authors declare no conflicts of interest.

## Data Availability

The datasets used and analyzed during the study are available from the corresponding author on reasonable request.
